# Riverside Greenway in Urban Environment: Residents’ Perception and Use of Greenways along the Huangpu River in Shanghai, China

**DOI:** 10.3390/ijerph18031120

**Published:** 2021-01-27

**Authors:** Zheng Zhao, Huimin Gan, Xin Qian, Jiahui Leng, Yanbin Wang, Peipei Wu

**Affiliations:** 1 College of Tourism, Shanghai Normal University, Shanghai 200234, China; zzshnu@shnu.edu.cn (Z.Z.); 1000464992@smail.shnu.edu.cn (X.Q.); 1000448702@smail.shnu.edu.cn (J.L.); 2 School of Economics and Management, Beijing Forestry University, Beijing 100083, China; hmgan143@bjfu.edu.cn; 3 Economic Development Research Center, National Forestry and Grassland Administration, Beijing 100714, China; 4 Shanghai Business School, College of Business Administration, Shanghai 200235, China

**Keywords:** greenway, resident perception, semantic differential scale, IPA model, Shanghai

## Abstract

Urban greenways improve green coverage rates in urban environments and transform these environments in a people-oriented manner. This study adopted semantic differential (SD) methods and an importance–performance analysis (IPA) model to evaluate resident perceptions and preferences of riverside greenways. A survey of 588 residents was conducted on typical natural greenways, built greenways, and mixed greenways along the Huangpu River in Shanghai. The results showed that resident perceptions of style, space, and distance differed markedly, whereas their perceptions of environmental and psychological characteristics were relatively similar. There were strong correlations between residents’ characteristics and their perceptions, especially for their perceptions of greenway style, sense of order, and distance from the river. By comparison, most residents preferred mixed greenways. Additionally, respondents from areas with natural and mixed greenways believed that they benefited, whereas those from areas with built greenways displayed a potential sense of deprivation. The results of IPA analysis provide further support for the above conclusions. As a whole, the relatively simple methods demonstrated here could be useful to quantitatively analyze the subjective perceptions of urban residents.

## 1. Introduction

Urban greenways have expanded urban leisure and service facilities worldwide. Urban residents, especially, can benefit from improved health, more opportunities for interactions with nature, and enhanced community cohesion. The concept of greenways originated from landscape science and was first proposed by William H. Whyte in 1959, while the following research gave a comprehensive definition of greenways from various aspects [1,2,3]. A greenway is a linear, natural, open space and is usually built along a natural or artificial channel, such as a riverside, valley, or road. It should contain exercise and recreational routes for pedestrians and cyclists, and connect features such as parks, green spaces, residential areas, fields, and various activity centers [4,5]. Currently, in major cities and megacities worldwide, various types of greenways, with reasonable planning and distinctive characteristics, play a pivotal role in the lives of residents, providing opportunities for interactions with nature despite fast-paced urban lives. First, urban greenways can gradually restore urban ecological environments damaged by human activities and play a vital role in purifying the air and conserving water and soil resources and other natural aspects [6,7]. Second, greenways can connect a series of fragmented natural and social elements in a linear form to promote the circulation of traffic, information, and personnel, thus maintaining and enhancing the ornamental and practical value of the areas along the greenway [8,9]. Finally, evidence suggests that greenway is among the most important factors for improving the surrounding environment and infrastructure, stimulating the revival of abandoned urban areas, furthering tap the development potential, and promoting the appearance of the city to achieve the coordinated development of urban social and economic development and ecological protection [10].

A major trend in the process of regional modernization and development is that populations are becoming concentrated from rural to suburban to urban areas. Therefore, in addition to considering the construction and productive functions during development, a city also needs to reserve space for a high quality of life for its urban residents. With the rapid development of modern cities, it is the core and fundamental purpose of future urban development to meet the residents’ desires for better city life. Over the past 40 years, with the rapid urbanization of China, the requirements of urban residents for better urban environments have also increased. In recent years, urban environmental problems have been continuously emerging. Many cities are facing problems of green spaces being restricted, the ecological environment being destroyed, and the urban environment appearing uniformly monotonous. For the healthy and sustainable development of large cities like Shanghai, it is especially important to create a “green ecological circle” to improve urban ecosystem functioning and the quality of life in the city.

In Shanghai, greenway construction primarily relies on natural and artificial corridors, such as green belts, forest belts, water channels, river networks, landscaped roads, and tree-shaded roads. Therefore, urban greenways are not isolated infrastructure and should not merely be placed in an open system. Instead, their relationships with residents’ lives should be completely considered [11,12]. At present, urban greenways have greatly improved the quality of life of urban residents, and the perceptions and feedback of residents about greenways are an important part of the functional improvement of greenways [13,14,15]. The construction of urban greenways in China began in 2009; the existing research on greenways is often focused on their macro benefits, while researchers have hardly dealt with evaluations based on the perspectives of users and studies on the relevant quantification of these perceptions are even scarcer. Indeed, contemporary urban residents’ perceptions of greenways have gradually expanded from traditional aesthetic and practical functions to deeper psychological and emotional satisfaction. Therefore, simplified mathematical and statistical models based on the objective characteristics of urban residents and greenways cannot perfectly explain the complex relationships between greenways and residents in real life. In other words, the positivist approach faces many obstacles in the analysis of current urban greenway-related issues. Therefore, a considerable portion of the research has extended into the “subjective world” of urban greenways, including “perceptual space” and “image maps” [16]. The essence of this trend is the integration of subjective judgment and objective feature perception [17]. Previous studies in many research fields have used semantic differential (SD) methods and usually combine them with other statistical methods [18,19]. Meanwhile, the importance–performance analysis (IPA) model first appeared in the commercial literature, whose theoretical basis is an expected uncertainty paradigm, which points out that public satisfaction is the product of the difference between expectation and performance [20]. Over time, it has formed a set of normative research paradigms, and has become one of the most common methodological tools in the entertainment and tourism literature [21,22]. Nowadays, the IPA model has been widely used in resources and environment-related research to help people understand the views of stakeholders on a range of issues, and some scholars have conducted relevant research on individual perceptions based on the IPA model [23,24]. These concepts and methods provide the basis for this study.

This study suggests that greenways are not only a means to improve the green coverage rate of urban environments, but they are also a method of transforming them in a people-oriented manner. Based on this understanding, evaluations were made on residents’ perceptions of riverside greenways through comparisons of three types of greenways along the Huangpu River in Shanghai. SD scales and an IPA model were used to comprehensively analyze the perceptions of residents with different location conditions and individual characteristics in response to spatial images based on three greenway types (natural, built, and mixed greenways). An objective of the research is to put forward suggestions on the improvement of different types of greenways from the perspective of residents through the conclusion of subjective analysis. Through this research, three unique contributions to the existing research are provided: the first contribution is that this study pays close attention to the perception factors related to urban greenways, and comprehensively considers the importance and performance of residents’ perception; the second contribution is that this study reports the significant differences among different types of greenways; hence, different types of greenways must be constructed according to the local conditions; the third contribution is that this study focuses on the subjective perception of residents, and further discusses the differences of their perception combined with their individual characteristics. In summary, this study deconstructs residents’ perceptions of urban greenways based on image elements and subjective image attributes and provides a theoretical basis for more reasonable and humanized urban greenway construction.

## 2. Materials and Survey Design

### 2.1. Study Areas and Observation Points

Shanghai is located in the Yangtze River Delta, east of China. Huangpu River is the main river crossing the urban area of Shanghai. The western bank of the Huangpu River is a historical and cultural building area with a long history; the east bank is a core financial area with skyscrapers, such as the Shanghai Center and the Shanghai World Financial Center. In recent years, a significant change in this area is reflected in the construction of greenway and other environmental facilities. After years of effort, Shanghai opened a waterfront public space on both sides of the Huangpu River in early 2018 and the 45 km waterfront area was transformed into a riverside greenway. The Huangpu riverside greenway is an outstanding representation of Shanghai’s urban greenway construction, as well as a good model of ecological priority development in contemporary large cities. Therefore, the Huangpu riverside greenway was selected as the research area.

**Figure 1:**
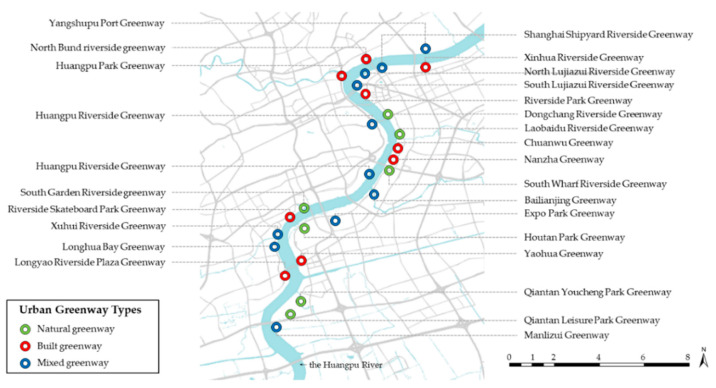
Map of the greenway along the Huangpu River, which is also the site of questionnaire distribution and data collection in this study.

According to Lynch’s (1960) theory, the urban image is the result of constant verification in the process of interaction with selected perceptual materials, and individual experiences in different environments will directly affect perception [25]. Therefore, it is necessary to further classify the research area. As this study aimed to increase understanding of public perceptions of urban greenways, the selected research sites were all representative riverside greenways located along the Huangpu River. Based on the original photographs taken during the field survey, the main greenways along the Huangpu River were classified into three types: natural greenways, built greenways, and mixed greenways. Previous studies have also extensively discussed the classification of waterfront greenways, which is also an important basis for the classification of greenways in this study [26,27]. The specific distributions of these are shown in Figure 1.

#### 2.1.1. Natural Greenways

**Figure 2:**
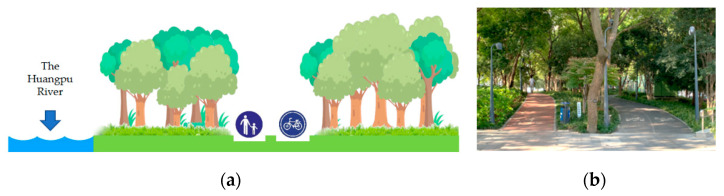
The natural form of greenway along the Huangpu River in Shanghai (natural greenway), seen from the perspective of (**a**) greenway designer and (**b**) greenway user, can effectively establish the organic connection between man and nature because the plants are luxuriant and the vegetation coverage rate is high along the natural greenway. Whereas, the routes of natural greenways are often uneven, with slopes and curves, and they are often narrow and crowded, causing the interference of citizens during sports or rest.

Because of the large number of residents and floating population in Shanghai, how to utilize and make the most of the limited green resources in urban landscapes, how to connect them, and how to achieve maximum ecological effects are the key and concerning topics in the construction of greenways. Natural greenways are the primary type of riverside greenway in Shanghai; they are also the most direct embodiment of the traditional concepts and modes of greenway development (Figure 2). It was obvious from the field survey that the plants along the natural greenways are flourishing, with high vegetation coverage, and this plays a role in enhancing and cooling the air. Some studies believe that greenways, as an important urban green infrastructure, can effectively establish an organic connection between man and nature and effectively improve the health of urban residents [28,29,30]. However, the routes in natural greenways tend to be uneven, with slopes and bends, and this makes it difficult for residents to control their breathing and rhythm during aerobic exercises such as jogging. Natural greenways are also often narrow and crowded, causing interference when exercising or resting. Additionally, some studies have pointed out that an overemphasis on green coverage may be detrimental to the maintenance of a good ecological environment [31,32]. Therefore, during the construction of natural greenways, it is necessary to highlight the natural elements and consider the practicability of each greenway. Moreover, many riverside spaces on both sides of the Huangpu River are occupied by built-up areas. Thus, the establishment and management of natural greenways would be difficult at present and in the future.

#### 2.1.2. Built Greenways

**Figure 3:**
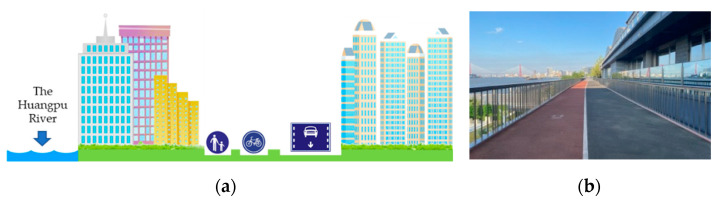
The built form of greenway along the Huangpu River in Shanghai (built greenway), seen from the perspective of (**a**) greenway designer and (**b**) greenway user, often have a wide sense of space and are combined with the construction of other municipal roads. On one hand, residents who exercise along these greenways have improved vision. On the other hand, built greenways are often affected by vehicle exhaust fumes, which reduce the air quality, and also the high-speed vehicles close to greenways reduce the safety coefficient for residents.

As the name implies, built greenways are greenways positioned in built-up areas. They are often extremely close to residential areas, business areas, and motorways. They are also the embodiment of the integration of greenways and modern urban areas (Figure 3). Built greenways have been developed in several areas that have not been fully utilized in the past (for example, the abandoned shipyards and factories along the Huangpu River), providing residents with active transportation alternatives, especially opportunities in built-up areas to walk, run, and cycle [33]. Although built greenways are quite different from traditional natural greenways in terms of green coverage, they still exhibit the functions of greenways in many respects [34]. Built greenways often have a wide sense of space and are combined with the construction of other municipal roads, while this form improves its utilization [35]. Along the way, there are seats, small squares, and direct drinking water points. There are several straight greenways, residents who exercise along these greenways have improved vision and consequently it is easy for them to accelerate during activities such as running. Additionally, because built greenways are close to municipal infrastructures such as urban motorways, bus stations, and subway stations, these greenways are easily accessible. However, built greenways also have inherent disadvantages; for example, they are often affected by vehicle exhaust fumes, which reduce the air quality. Besides, high-speed vehicles close to greenways reduce the safety coefficient for residents. In general, although clean and modern built greenways have certain advantages, they often lack the natural and ecological functions of traditional greenways [36,37].

#### 2.1.3. Mixed Greenways

**Figure 4:**
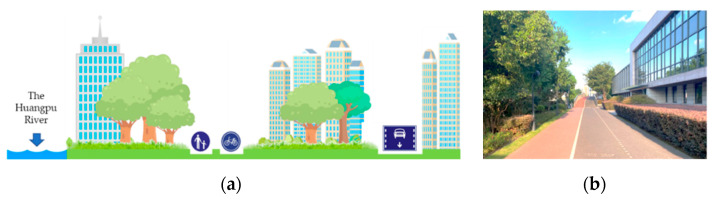
The mixed form of greenway along the Huangpu River in Shanghai (mixed greenway), seen from the perspective of (**a**) greenway designer and (**b**) greenway user, combines the characteristics of natural and built greenways, which are integrated into the urban landscape as part of the daily infrastructure with which residents interact. They integrate the ecological benefits of green plants into the built-up areas of cities as much as possible, so that residents can enjoy the conveniences offered by modern cities, concurrently also experiencing the feeling of returning to nature.

City development should be human-centric, and the construction of greenways should also have the organic unity of nature and society as its core. For urban built-up areas that were constructed with reinforced concrete, the importance of the ecological environment is more prominent. Mixed greenways combine the characteristics of natural and built greenways, it adapts to the more practical needs of residents and has a stronger interaction with them [13,14]. Mixed greenways are integrated into the urban landscape as part of the daily infrastructure with which residents interact. The mixed greenways have advantages over both natural and built greenways (Figure 4). They integrate the ecological benefits of green plants into the built-up areas of cities as much as possible, so that residents can enjoy the conveniences offered by modern cities, while concurrently also experiencing (however, moderately) the feeling of returning to nature [35]. From this perspective, mixed greenways have the advantage of practicality. In contrast, there are many difficulties involved in the construction and management of mixed greenways. For example, it is extremely difficult to construct mixed greenways in Shanghai as they need to be squeezed into existing built-up urban areas. Nevertheless, in the long run, the convenience of urban life and the beauty of the natural environment should be coexistent and complementary, not the opposite. Undoubtedly, the harmonious integration of nature and modernity is the most suitable form of urban greenway construction.

By examining the real-life conditions of riverside greenways in Shanghai, it can be observed that the three abovementioned greenway types encompass all the riverside greenways along the Huangpu River, which are representative and typical of the research area. Furthermore, it should be noted that, on both the east and west banks of the Huangpu River, the footpath is located on the side nearest the river, while the cycle path is located on the other side. Moreover, this study uses the form “footpaths on the left, cycle path on the right” to avoid visual confusion.

### 2.2. Data Collection

Based on existing research and established questionnaire design norms and methods, the questionnaire for this study was designed in three steps. First—through literature analysis, expert interviews, and topic group discussions—the first draft of the questionnaire was prepared. Second, through pre-survey activities, issues related to the reliability and validity of the questionnaire were determined and resolved to improve the overall quality of the questionnaire. Third, the questionnaire was revised to produce the final draft (see Appendix A). On this basis, combined with the real-life conditions of the Huangpu River waterfront area, seven natural greenways, nine built greenways, and 11 mixed greenways were selected to conduct the questionnaire survey. During each on-the-spot investigation, the questionnaire was continuously revised on the premise of ensuring overall consistency. The core principle of questionnaire design and improvement is efficiency and comprehensiveness. The purpose is to make the questionnaire as simple and clear as possible to accurately obtain the data needed for the study and to improve the response rate and effectiveness of the survey. To achieve the aim, existing studies were referred to on improving questionnaire responsiveness [38,39]. According to practical research experience, the average completion time of a comprehensive and high-quality questionnaire is approximately 35–45 min.

Based on the research areas, questionnaires, corresponding survey methods, and field surveys were conducted in August 2020. Data were collected along the three types of greenways through intercept surveys of residents and survey sessions were scheduled by using a stratified random sampling protocol to ensure adequate numbers of respondents. As for the selection of respondents, a survey was conducted within the greenway and its surrounding areas, because the respondents here will have a relatively clear perception of the greenway, to improve the quality of research data. On the other hand, considering that some residents have difficulties in expressing and answering questions, the survey was conducted face-to-face. The investigators gave appropriate explanations to the questions that may arise in the questionnaire, to ensure the smooth progress of the survey. The investigators were all graduate students with professional academic backgrounds and field investigation experience. The investigators underwent systematic training on research methods and data collection before carrying out the surveys. Overall, 633 questionnaires were completed, of which 588 were validated. The overall effective response rate was 92.89%.

## 3. Methods and Model Design

### 3.1. Semantic Differential Methods

Surveying the behavioral preferences of respondents has always been an important means of studying urban spatial perception [40]. The SD method is a psychological research method proposed by Osgood (1957). The benefit of the method is that it is easy to operate and can be used objectively and quantitatively to analyze the research object while avoiding the disadvantages of the urban image method. The SD method has been used in many research fields and is often combined with other statistical methods [41]. The macro-level includes research on the overall urban space, and the micro-level includes research on architectural space and street space [42]. The SD method assumes that human beings attach extensive and common emotional meanings to concepts or words and that these do not change significantly with cultural and linguistic differences. Therefore, it is reasonable and effective to ask respondents directly about such concepts. However, the range of activities of different resident groups will differ under the influence of individual characteristics, preferences, and other objective factors. Thus, their perception of urban greenways will also differ. It is generally believed that perceptions, utilization, and preferences concerning urban greenways are affected by individual functional and structural variables (occupation, age, marriage, etc.); social, cultural, and political variables (income, social class, etc.), and spatial variables (residential area, distance, etc.).

In summary, based on relevant existing research, 12 pairs of adjectives were selected to describe the characteristics of urban greenways and constructed an SD scale according to residents’ perceptions to reflect the psychological feelings of the respondents regarding urban greenways [8,43,44,45,46]. Specifically, six evaluation elements and 12 evaluation items were identified. Each evaluation item corresponded to a pair of adjectives, which were the evaluation factors in this study. The specific SD scale is shown in Table 1. The evaluation items (categories) provide a clear expression of the evaluation elements (variables) which were selected by combining the characteristics of greenways in Shanghai and the cognitive ability of its residents.

Based on the evaluation factors listed in Table 1, the evaluation scale was classified into five levels with five intervals between each adjective combination. These intervals were symmetrical, with 0 as the midpoint. The interval values were −2, −1, 0, 1, and 2 from left to right, and these were used as the scoring method for the resident’s evaluations. The higher the score of each evaluation item, the more inclined the evaluation factor is to the right-hand adjective; the lower the score, the more inclined the evaluation factor is to the left-hand adjective.

### 3.2. Importance–Performance Analysis

There are several methods to measure perception and preference. Similar to previous studies, an IPA model was adopted to analyze and measure residents’ comprehensive perception of urban greenways, identify existing problems, and find specific targets to improve residents’ satisfaction. The relevant data was obtained through questionnaire survey [47]. Of course, this method has defects, and relevant research has also conducted corresponding discussions in this regard [48].

**Figure 5:**
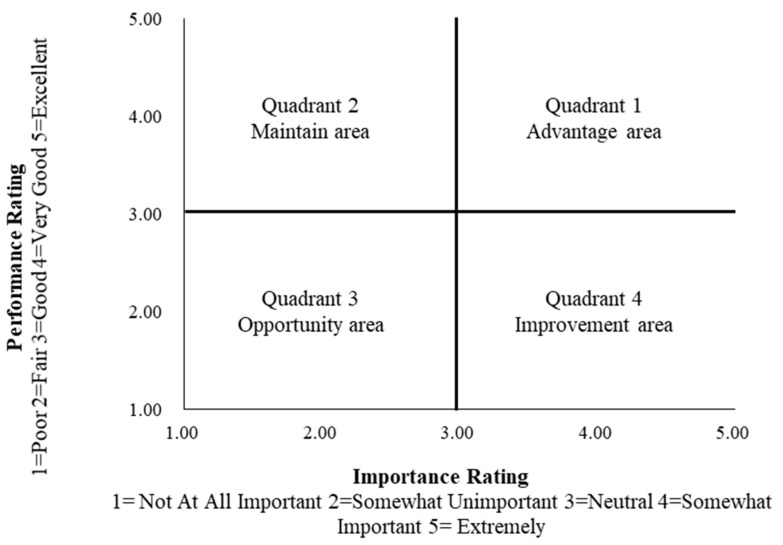
Scheme of importance–performance analysis grid.

The IPA model is divided into four quadrants with performance and importance as the axes [21,49]. Through the quadrant analysis of performance and importance, the results of residents’ comprehensive perception can be classified as follows: (1) Quadrant 1, in the upper right corner, is the advantage area. The residents believe that factors in this area are very important and the performance of the urban greenway is very good; (2) Quadrant 2, in the upper left corner, is the maintenance area. The residents believe that the importance of the factors in this area is low, while the performance of the urban greenway is good. Therefore, keeping these factors stable can be beneficial; (3) Quadrant 3, in the lower-left corner, is the opportunity area. The residents believe that the factors in this area are not particularly important and the performance of the urban greenway is relatively poor. However, it does not mean that urban greenway construction can neglect the factors in this area; instead, special attention is required on the cause analysis and breakthrough points should be identified to improve residents’ satisfaction; (4) Quadrant 4, in the lower right corner, is the improvement area. The residents believe that the factors in this area are extremely important, while the performance of the urban greenway is not equally good. More attention should be paid to the planning and management work in the future, and focus should be placed on repairing and improving these factors. In this study, a five-point Likert scale was used to measure residents’ perception of urban greenway importance and performance, as shown in Figure 5.

## 4. Results

### 4.1. Demographic Characteristics of the Respondents

Regarding individual characteristics (shown in Table 2), the proportions of male and female respondents differed slightly between the different types of greenways but were generally balanced. More than 75% of respondents were under 40 years old, and respondents from the natural greenways were relatively older. More than 80% of the respondents had high school or higher educational backgrounds. The proportion of unmarried respondents from the built greenways was slightly higher than those from the natural or mixed greenways. The overall family size of respondents was three, of which the average number of elderly people and children was one or fewer. Fewer respondents were registered as Shanghai residents than those who were not registered, and the numbers of people who had lived in Shanghai for five years and more than fifteen years were large. Approximately 60% of residents worked 20–50 h per week. In terms of economic conditions, more than 80% of respondents had per capita monthly incomes below ¥12,000. Furthermore, more than half of the respondents lived within the Middle Ring Road, which was consistent with the expected number and the population density of residents in the core urban area of Shanghai. At the same time, it also ensures the quality of the respondents in the survey site and their understanding of the research topic. Additionally, for more than 60% of the respondents from the built and mixed greenways, the nearest greenway to their home was within 5 km. For the natural greenway respondents, this proportion exceeded 70%. Therefore, the primary method of traveling to the greenway was walking, and these two characteristics were interlinked.

### 4.2. Differences in Residents’ Perceptions

To reveal the tradeoffs in residents’ perceptions of different types of greenways and to explore residents’ needs concerning these types of greenways, the SD method was used. The SD factor scores of the three types of greenways are shown in Table 3. Overall, the perception differences among the three greenway types for style, space, and distance were relatively large, whereas those for environment and psychology were relatively small. The results of a one-way ANOVA showed that the perception differences among the evaluation factors for the three greenway types were all highly significant.

**Figure 6:**
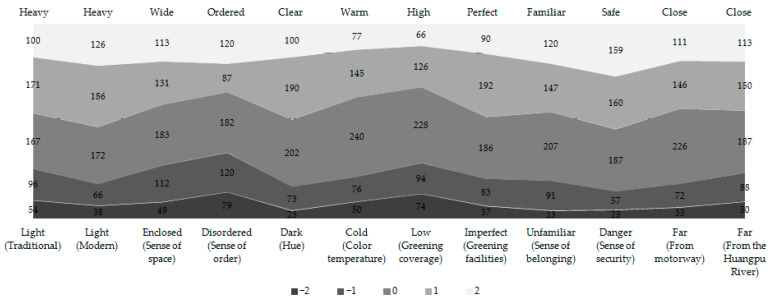
Frequency distribution of semantic differential evaluation elements of the Huangpu riverside greenways.

Based on their SD factor scores, the public perception of each greenway evaluation element can be quantified and graded. The frequency characteristics of the different items were explored and, then, the residents’ overall perceptions were analyzed (Figure 6). The vast majority of the respondents had perception evaluations between −1 and 1. According to the frequency distribution of the evaluation factors, there were 10 obvious trends in the perception of the Shanghai Huangpu riverside greenways: greenways are heavily traditional and spatially wide, brightness and contrast are clear, overall color temperature is warm, greening facilities are perfect, there is a sense of belonging and familiarity, greenways are safe, and greenways are close to both the motorway and the Huangpu River. However, the perception trends of the other two perceptual elements were not obvious. These results reflect the overall perceptions of all the respondents to the elements of the Huangpu riverside greenways.

**Figure 7:**
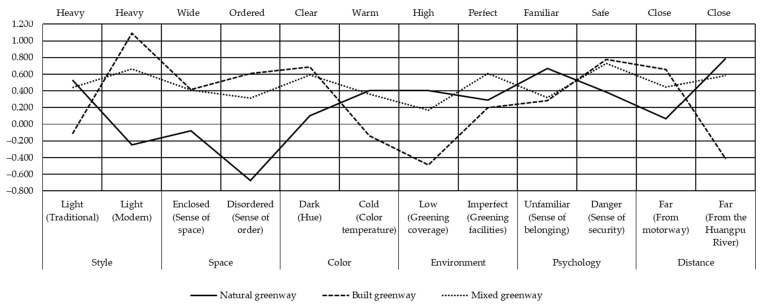
Semantic differential evaluation curve of different greenway types.

Similarly, corresponding SD evaluation curves (Figure 7) can be drawn to show the perception differences of different types of greenways more intuitively to reflect the characteristics, advantages, and disadvantages of each type [50].

#### 4.2.1. Style

The advantage of natural greenways is that residents can have a strong sense of traditional style, which is an invaluable asset in large modern cities. Accordingly, the public’s perception of the traditional style of built greenways is weak, and it is replaced by a strong sense of modern style. In contrast, both traditional and modern styles are obvious in mixed greenways as they integrate the two styles. With this integration, the residents’ perception of both traditional and modern styles has been highlighted. The above conclusions about greenway style perceptions are closely related to the inherent characteristics of the different types of greenways, which can now be better understood. Besides, what requires our attention is whether different greenway styles can be well integrated with their location to ensure that all of the potential functions are fully utilized.

#### 4.2.2. Space

People’s perception of the spatial sense and orderliness of natural greenways is weak. This is because natural greenways have a relatively high amount of vegetation coverage, resulting in a relatively closed and disordered feeling. In contrast, built and mixed greenways are characterized by a broad and orderly feeling. In general, the support services of built and mixed greenways are better, ranging from setting pedestrian and cycling lanes to the operation of public toilets, all of which have their own detailed standards, and the corresponding management practices are also detailed. From this perspective, built and mixed greenways have certain advantages. While other studies have reported that a considerable number of residents prefer disordered and primitive natural areas.

#### 4.2.3. Color

In recent years, the concepts and methods of greenway construction in Chinese cities have gradually matured. Greenway construction in Shanghai attaches great importance to greening, colorization, rarity, and benefit, especially concerning rich vegetation levels and the diversity of vegetation types. In this regard, owing to the better vegetation condition and higher canopy density, the hue of natural greenways is darker than those of the other two greenway types. In terms of color temperature, there are more flowering plants and shrubs along natural greenways. These colorful plants give the residents a warm feeling. In contrast, built greenways are constructed on urban built-up areas and their color temperature is relatively cold. As far as mixed greenways are concerned, they attempt to integrate more natural elements into built-up areas; hence, the warmth of their color temperatures is improved. In contrast, greenway roads themselves can be a source of color. For example, a red footpath can promote feelings of joy and vision, whereas a gray cycle path can promote feelings of openness and progress. At different times of the day, the greenway interacts with climate, temperature, light, and other factors, prompting different reactions from the public.

#### 4.2.4. Environment

It is obvious that vegetation coverage is the highest in natural greenways, followed by mixed greenways and built greenways. For the construction and preservation of natural elements during greenway construction, the original natural resource base of Shanghai has been completely utilized. The urban ecosystem has been preserved as far as possible, and ecological forests, street gardens, and urban green spaces are connected by greenways. In terms of green facilities, the three types of greenways give residents a feeling of perfection. According to the survey, the current mixed greenways have the highest degree of specialization, especially regarding service facilities such as seats, public toilets, and lighting. Mixed greenways make full use of existing facilities to minimize the construction activities in green areas. Because of the need to preserve the traditional look of greenways as far as possible, the setting up of green facilities in natural greenways is restricted; while their performance is still fair. In contrast, there is room for built greenways to have an improvement in the construction of green facilities.

#### 4.2.5. Psychology

The sense of belonging of residents directly affects their perception, evaluation, and utilization of greenways. At present, people most often report a sense of belonging in natural greenways, whereas their sense of belonging in the other two types of greenway remains relatively low. This is related to the psychological feelings brought about by the natural environment. The field survey also provided an interesting piece of evidence; during greenway construction, a large number of illegal buildings and structures along the Huangpu River were demolished and replaced with greenways; additionally, the existing vegetation was preserved by changing the alignment and shape of the greenway. Most of the construction and renovation decisions concerning the greenways were decided by the residents around the greenway who participated in the implementation. These decisions, therefore, reflect the most vital requirements of the residents and provide them with a great sense of belonging. Corresponding to the sense of belonging is the sense of security felt in the greenways. The results show that built and mixed greenways give residents a higher sense of security. According to the interviews, most Shanghai residents have been living in a modern and noisy urban environment for a long time. Built and mixed greenways offer them a sense of security, whereas the relatively closed natural greenways promote mild feelings of loneliness and uneasiness. However, some respondents suggested that this feeling will gradually decrease with the passage of time and the continuous improvement of greenways.

#### 4.2.6. Distance

This is determined by the objective location conditions of the riverside greenway in Shanghai and is also an inherent characteristic of greenways in the traditional sense. When residents engage in activities such as exercise, recreation, and entertainment, the sense of distance between them and the natural environment is narrowed. In contrast, the built and mixed greenways are close to the motorway; many sections of the built greenway are close and parallel to the motorway. The built greenway is located far from the Huangpu River and is a typical greenway type in built-up areas. Based on the basic conditions of these two types of greenways, the distances between the mixed greenway and the motorway and Huangpu River are moderate. The opinions differ among the residents and scholars regarding this point. The sense of distance is not only physical but also psychological. Far or near?—this is a topic worthy of debate and perhaps the best answer would that both far and near greenways have their own advantages and disadvantages.

As for the possible correlations between residents’ perception of greenways and their individual characteristics, a correlation coefficient table of the 12 types of resident perceptions against their 14 individual characteristics was constructed (Table 4). Except for education status, family size, and residential area, the individual characteristics of the residents correlate with their perception evaluations; correspondingly, there is also the possibility that some functional characteristics of greenways cannot be perceived by residents. Specifically: (1) average working hours per week, per capita monthly income, and distance from home to the nearest greenway exhibited the strongest correlations with greenway perceptions. Therefore, in terms of greenway construction in Shanghai, special attention should be paid to the different requirements of residents with different working hours and income levels and who live at different distances from the greenway. Besides, in Shanghai, as a city with strong inclusiveness and openness, the perception of residents about urban greenways does not differ significantly with differences in education status. Moreover, with the continuous expansion of the scope of greenway construction, the correlations between residents’ perceptions and their family size and resident area are no longer obvious. (2) In terms of residents’ perceptions of greenways, the relationships among their perception of traditional style, sense of order, and distance from the Huangpu River are the strongest. The perception of Shanghai residents regarding greenways is still focused on the visual appeal, and the residents are mostly concerned with the location selection of riverside greenways.

### 4.3. Differences in Residents’ Preferences

Based on the three types of greenways, this study used the five-point scale method and two questions to investigate residents’ preferences concerning greenways. First, each respondent chose their most preferred greenway among the three types of greenways and made an overall comparison among them. Second, each of the respondents rated each type of greenway from 1 to 5 (weak to strong) to measure the specific degree of preference of each resident. It should be noted that when the respondents chose and rated the three types of greenways, the different types of greenways were assumed to exist independently. The selection and scoring by residents are shown in Table 5. It can be seen that 41.50% of the residents preferred mixed greenways, 34.18% preferred natural greenways, and only 24.32% preferred built greenways. As for the preference scores, the overall average score of natural greenways was 4.066, which was higher than that of built greenways (3.311), but lower than that of mixed greenways (4.112). Based on this, it is believed that mixed greenways are the favorite of the residents as they not only provide convenient conditions for modern urban life but also integrate natural elements, improving the ecological environment and the well-being of the residents.

Differences were also analyzed in greenway perception among different resident groups, that is, the perception of residents within a certain type of greenway for all three types of greenway, and cognitive matrixes were obtained by comparing their perception differences (Table 6). It can be seen from the matrix results that respondents from mixed greenway had the most positive overall evaluation, followed by respondents from natural greenways. The lowest results were from respondents from built greenways. Respondents from natural and mixed greenways had the highest perception of greenways (4.066 and 4.112, respectively), whereas respondents from built greenways had the lowest (3.311) perception. On the one hand, this shows that residents prefer mixed greenways with both natural and social characteristics. On the other hand, respondents from natural and mixed greenways were satisfied with the greenways, whereas respondents from built greenways preferred the other two types of greenways. In other words, at present, the built greenways do not satisfy the requirements of the public and need to be further improved through the integration of natural elements.

**Figure 8:**
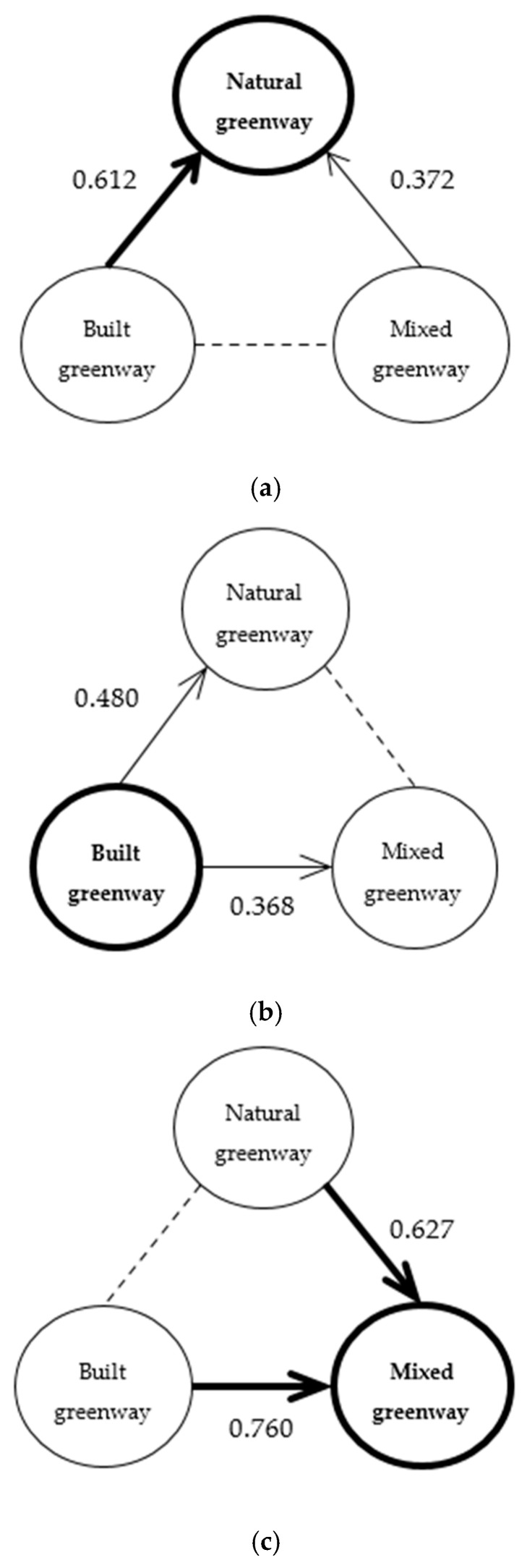
Comparison of greenway perception among different resident groups. Consistent with existing research results, the arrows in this figure point toward the beneficiaries and away from the injured parties. Besides, coarse arrows represent paths with relatively strong resident perceptions [50]. Thus, respondents from (**a**) natural greenways and (**c**) mixed greenways believe themselves to be beneficiaries; Respondents from (**b**) built greenways believe that they belong to the injured party.

It can be seen from the Figure 8 that (1) respondents from natural greenways believe themselves to be beneficiaries. They are relatively satisfied with the utility of their greenways, largely owing to the objective characteristics of natural greenways. Thus, natural greenways can easily meet the needs of the public because they can adapt to the core demands of residents in greenways. (2) Respondents from built greenways believe that they belong to the injured party and have a sense of deprivation regarding the utility of their greenways. The reason for this is that, although the modernization of built greenways is better than that of natural and mixed greenways, their natural characteristics are relatively weak. With the continuous improvement of the ecological environment demand of Shanghai residents, more natural elements should be integrated into the construction of built greenways in the future to meet the needs of the residents. Thus, a transition to mixed or natural greenways should be made, as far as possible. (3) Respondents from mixed greenways also believe that they are beneficiaries. They are relatively satisfied with the utility of their greenways. Because mixed greenways have both natural ecological and social functions. Here, residents can not only enjoy the natural aspects of greenways but also benefit from the convenience of modern urban construction. Therefore, the comprehensive perception of residents regarding mixed greenways is relatively good.

### 4.4. Differences in Importance–Performance Scores of Residents

**Figure 9:**
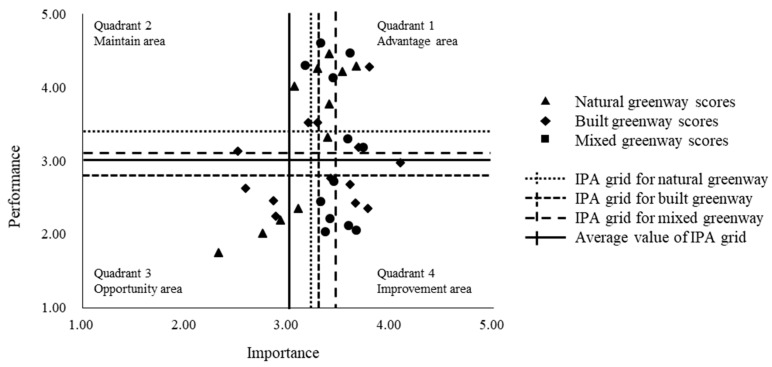
Importance–performance analysis grid of the three types of urban greenways.

The perceptions and preferences of residents for urban greenways are based on their evaluations of the importance and performance of greenway factors. An IPA grid was prepared to illustrate the average importance and performance values of each greenway factor and differences among the different greenway types, as shown in Table 7 and Figure 9.

According to the 12 SD factors, the corresponding points of each urban greenway type were drawn on an IPA grid. The residents had similar perceptions regarding green facilities and a sense of belonging in the different urban greenways and these perceptions exist in Quadrant 1. Thus, these two factors are very important, and the performance of urban greenways is also very good [35,47]. Most of the studied factors are very important to natural greenways and their corresponding performance is also very good. In contrast, there is still room for improvement for the other two types of greenways. Residents believe that a modern style, sense of space, and sense of order have low priority for natural greenways; hence, they care little about these factors. Concurrently, residents believe that these factors are very important in built greenways; while their performance is not satisfactory at present and they have not met expectations. This can be taken as a key repair and improvement objective in future planning and management practices. Additionally, residents believe that traditional style, color temperature, and distance from the Huangpu River are irrelevant in relation to built greenways; these factors are the advantages of natural greenways, which show that the importance–performance perception of residents is closely related to the construction style and location characteristics of greenways. Finally, although the public believes that the importance of green coverage is low for built greenways, they are performing well at present. Thus, maintaining the green coverage of built greenways is very important; while it should also be made sure that the green coverage of built greenways is not oversupplied, a point that has been discussed previously [31,32].

## 5. Discussion

Urban greenways connect the city and natural environments, integrating traditional and modern styles. Greenways are not only a means to improve the green coverage rate of urban environments but also a way of transforming them in a “people-oriented” manner. In other words, in the process of urban greenway development and improvement, it is most important to consider urban residents and meet their requirements for a higher quality of life. In this sense, urban greenways are not merely roads but also an idea of healthy urban development. Based on this understanding, this study pays attention to importance and performance at the same time and puts forward targeted improvement and promotion suggestions, it provides three unique contributions to the existing studies.

The first contribution is that this study pays close attention to the perception factors related to urban greenways, and comprehensively considers the importance and performance of residents’ perception. Regarding the perceptions of residents, Shanghai greenway projects should focus on the traditional style, sense of order, and distance from the Huangpu River, as these factors had the strongest correlations with the individual characteristics of residents and were the most important aspects in overall greenway perceptions. Therefore, special attention should be paid to these factors during greenway construction and improvement. On the whole, greenways in their pure natural form, sufficient green coverage, or strong modern elements by themselves cannot bring real satisfaction to residents. The perceptions of residents need to be enhanced through the integration of multiple sensory effects and various sensory elements. On the one hand, the construction of greenways in Shanghai cannot be separated from the social, economic, and cultural aspects of the city. The coupling of ecological and social benefits should be realized; more natural elements should be included, and attention should be paid to the synchronous improvement and promotion of modern urban service functions and the urban ecological environment. On the other hand, as a complex socio-economic and ecological environmental system, several factors need to be considered holistically during greenway construction. It should not only consider the restoration of urban nature but also ensure the normal operation of urban social service functions.

The second contribution is that this study reports the significant differences among different types of greenways; hence, different types of greenways must be constructed according to the local conditions. Since the overall design of open space should consider the accessibility, continuity, and functionality of the space, while reflecting on the characteristics and advantages of various greenways, it should also consider the coordination and unity among various greenways to ensure that there is an orderly and reasonable transition in space. In addition, it is necessary to highlight the advantages of the different types of greenways and consider the needs of different groups to ensure that all greenways can be used effectively by the public. According to a concept provided by the Japanese architect Kurokawa Kisho, “symbiosis” suggests that a “symbiosis” between humans and landscapes is the ultimate goal, and the only way to achieve this goal is to obtain the identity of the experience subject and the landscape object. In this study, each of the three greenways had its own characteristics; however, people’s understanding of these greenways was often from the perspective of practical benefits and functionalism. That is to say, residents’ perceptions of greenways were relatively superficial. Therefore, in the process of constructing and improving greenways, planners should consider the external performance and internal functions of greenways to meet the multi-dimensional requirements of the residents.

The third contribution is that this study focuses on the subjective perception of residents, and further discusses the differences in their perception combined with their individual characteristics. Urban greenways are like mirrors that reflect the comfort and civilization of a city. Greenways provide a brand-new interactive platform for the city and its residents. In this regard, Shanghai greenways should consider the different requirements of residents with different working hours and income levels, and who live at different distances from the greenway. In addition, the construction and improvement of urban greenways cannot be separated from the attention and extensive participation of residents. Therefore, on the one hand, it is necessary to strengthen the publicity work related to urban greenways and deepen the perception levels of residents regarding greenways. On the other hand, it is also necessary to allow residents to participate in the construction of urban greenways as much as possible so that they can provide suggestions for the continuous improvement of these greenways; this would help urban greenways truly reflect the demands, preferences, and needs of the residents.

This study used SD scales and an IPA model to quantitatively analyze the subjective perceptions of residents in a relatively simple manner. However, considering that urbanization and urban ecological construction in Shanghai are constantly changing and improving, greenway research on the perceptions of residents needs to be constantly supplemented and improved. For example, the types of greenways in Shanghai could be further subdivided and the research areas could also be expanded or focused to draw more targeted conclusions.

## 6. Conclusions

This study evaluated the residents’ perceptions of riverside greenways based on the comparison of three types of greenways along the Huangpu River in Shanghai. SD scales and an IPA model were used to comprehensively analyze the perceptions of residents based on three types of greenways (natural, built, and mixed greenways), location conditions, and individual characteristics. The importance–performance perception of residents was also analyzed and compared among the three greenway types. The goal of the study is to put forward suggestions on the improvement of different types of greenways from the perspective of residents through the conclusion of subjective analysis. The results show that this study deconstructs residents’ perceptions of urban greenways based on image elements and subjective image attributes, and provides a theoretical basis for more reasonable and humanized urban greenway construction.

Specifically, the perceived differences between the different types of greenways, in terms of style, space, and distance, were relatively large, whereas those concerning environmental and psychological characteristics were relatively small. Moreover, the differences in the perception of various aspects of greenways were obvious, which were closely related to their characteristics. Specifically, residents had a strong sense of traditional and modern styles of natural and built greenways, whereas their perception of mixed greenways combined the two. Excluding education status, family size, and residential area, the individual characteristics of the residents exhibited certain correlations with their perceptions.

Additionally, among the three types of greenways, the residents preferred the mixed greenways the most, followed by the other two types of greenways. In terms of the perception of different types of greenways, respondents from natural and mixed greenways had the highest perception of the corresponding greenway types, while respondents from built greenways had the lowest perception. This indicates that respondents from built greenways prefer the other two types of greenways. Similarly, respondents from natural and mixed greenways saw themselves as beneficiaries; the comprehensive perceptions of these respondents were relatively positive. Respondents from built greenways believe that they are the injured party; they have a sense of deprivation about the utility of their greenways and, thus, it is necessary to appropriately incorporate natural elements into the construction of built greenways in the future.

The IPA results show that residents generally have a good perception of the importance and performance of different types of urban greenways and green facilities and feel a sense of progress. For natural greenways, factors such as modern style, sense of space, and sense of order do not seem to be important. The public believes that these factors are very important for built greenways, however, these have not met the performance expectations of residents. Residents also believe that traditional style, color temperature, and distance from the Huangpu River are irrelevant to built greenways; while these factors are advantages of natural greenways. Finally, although the public rated the importance of green coverage in built greenways as low, they has performed well at present. In other words, for built greenways, green coverage is not particularly important. Overall, IPA provides an effective and flexible tool for assessing residents’ perceptions of urban greenways and a basis for the planning and management of urban greenways.

## Figures and Tables

**Table 1:** The semantic differential scale of residents’ perceptions of riverside greenways.

Category	Symbol	Variable	Index	Definition
M1	Style	Traditional	Light–Heavy	Traditional degree of greenway
M2		Modern	Light–Heavy	Modern degree of greenway
M3	Space	Sense of space	Enclosed–Wide	Spatial looseness and intensity of greenway
M4		Sense of order	Disordered–Ordered	Orderliness of greenway
M5	Color	Hue	Dark–Clear	Brightness and contrast of greenway
M6		Color temperature	Cold–Warm	Overall cool and warm degree of greenway
M7	Environment	Vegetation coverage	Low–High	Vegetation coverage of greenway
M8		Greening facilities	Imperfect–Perfect	Greening facilities along the greenway
M9	Psychology	Sense of belonging	Unfamiliar–Familiar	Sense of belonging and familiarity of greenway
M10		Sense of security	Dangerous–Safe	Public security perception of greenway
M11	Distance	From motorway	Far–Close	Distance between greenway and motorway
M12		From the Huangpu River	Far–Close	Distance between greenway and the Huangpu River

**Table 2:** Descriptive statistics of samples (%).

Variable	Natural Greenways	Built Greenways	Mixed Greenways	Variable	Natural Greenways	Built Greenways	Mixed Greenways
(X1) Gender				(X9) Residence in Shanghai			
Men	45.92	55.10	43.88	<1 year	32.14	36.22	26.02
Women	54.08	44.90	56.12	1–5 years	22.96	21.94	20.92
(X2) Age				5–10 years	9.18	7.65	7.65
Age 17 and under	3.57	6.63	5.10	10–15 years	8.67	6.63	5.61
18–29 years old	56.63	63.78	41.84	15 years or more	27.04	27.55	39.80
30–39 years old	16.33	13.78	20.92	(X10) Average working hours per week			
40–49 years old	5.61	7.14	17.35	<1 h	16.33	18.88	14.80
50–59 years old	6.63	2.04	7.14	1–20 h	13.27	13.27	12.24
Age 60 and over	11.22	6.63	7.65	20–40 h	26.53	26.53	24.49
(X3) Education status				40–50 h	36.73	33.16	45.41
Primary school and below	2.04	2.04	1.02	>50 h	7.14	8.16	3.06
Junior middle school	13.27	6.63	10.71	(X11) Per capita monthly income			
High school	19.39	28.06	24.49	¥4000 and below	37.24	36.22	29.08
Undergraduate	45.92	50.51	52.55	¥4001–¥8000	34.69	32.14	34.69
Master’s degree and above	19.39	12.76	11.22	¥8001–¥12,000	12.76	16.33	15.82
(X4) Marital status				¥12,001–¥16,000	7.14	7.65	8.67
Unmarried	58.16	65.82	45.41	¥16,001–¥20,000	1.53	4.08	3.06
Married	41.84	34.18	54.59	>¥20,000	6.63	3.57	8.67
(X5) Family size				(X12) Residential area			
1 person	11.22	10.20	9.69	Within Inner Ring Road	22.45	28.06	30.10
2 persons	15.82	16.84	21.43	Inner–Middle Ring Road	32.14	29.59	28.06
3 persons	35.71	40.82	38.78	Middle–Outer Ring Road	22.45	17.86	24.49
4 persons	22.45	18.37	14.29	Outer Ring Road–The Ring Expressway	12.24	11.73	8.16
5 persons or more	14.80	13.78	15.82	Beyond the Ring Expressway	10.71	12.76	9.18
(X6) Number of elderly people				(X13) Distance from home to the nearest greenway			
1 person or less	70.41	72.96	77.55	<1 km	39.29	35.20	26.02
2 persons	22.45	20.41	16.33	1–5 km	32.14	30.61	36.22
3 persons or more	7.14	6.63	6.12	5–10 km	10.71	13.78	22.96
(X7) Number of children				>10 km	17.86	20.41	14.80
1 person or less	90.31	91.33	92.86	(X14) Main modes of transportation			
2 persons	8.16	7.14	6.12	Self-driving	11.22	10.20	16.84
3 persons or more	1.53	1.53	1.02	Walking	49.49	47.45	45.41
(X8) Shanghai residence registration status				Motorcycle and bicycle	10.71	15.31	12.76
Yes	36.73	34.18	47.96	Taxi	1.53	1.53	1.02
No	63.27	65.82	52.04	Bus and subway	27.04	25.51	23.98

**Table 3:** Semantic differential factor scores of residents’ perceptions of riverside greenways.

Variable	Natural Greenways	Built Greenways	Mixed Greenways	Overall	F
M1	0.531	−0.117	0.439	0.284	17.901 ***
M2	−0.245	1.092	0.663	0.503	92.681 ***
M3	−0.077	0.418	0.408	0.250	11.146 ***
M4	−0.673	0.607	0.316	0.083	62.850 ***
M5	0.102	0.689	0.592	0.461	19.182 ***
M6	0.403	−0.138	0.362	0.209	15.573 ***
M7	0.403	−0.490	0.168	0.027	35.316 ***
M8	0.291	0.199	0.607	0.366	7.659 **
M9	0.668	0.286	0.321	0.425	7.465 **
M10	0.388	0.776	0.730	0.631	7.338 **
M11	0.066	0.658	0.449	0.391	15.444 ***
M12	0.791	−0.413	0.582	0.320	71.082 ***
Average	0.221	0.297	0.470	0.329	-

***, **, and * represent significance levels of 1, 5, and 10%, respectively, and the variables M1–M12 are defined in Table 1.

**Table 4:** Correlation table between resident perceptions and individual characteristics.

	Variable	M1	M2	M3	M4	M5	M6	M7	M8	M9	M10	M11	M12
Characteristics													
X1		0.059	−0.027	−0.045	−0.032	−0.089 *	−0.014	−0.069	−0.044	−0.100 *	0.022	−0.034	0.017
X2		0.106 *	−0.101 *	−0.036	−0.056	−0.055	0.049	0.045	−0.010	−0.024	−0.006	−0.021	0.090 *
X3		−0.062	0.016	0.024	−0.008	0.067	−0.044	−0.069	−0.027	−0.035	0.032	0.049	0.000
X4		0.096 *	−0.054	0.009	−0.014	−0.010	0.023	0.060	0.011	0.010	−0.002	0.037	0.081 *
X5		0.017	0.025	0.030	−0.033	0.033	−0.022	−0.052	0.059	0.020	0.000	0.003	−0.016
X6		−0.043	−0.027	−0.023	−0.044	0.029	0.100 *	−0.078	−0.005	−0.004	0.056	−0.026	−0.012
X7		−0.082 *	0.008	−0.006	−0.030	−0.032	−0.033	−0.050	0.090 *	0.056	−0.016	−0.081	0.015
X8		−0.072	−0.030	−0.028	−0.122 **	−0.002	−0.068	−0.065	−0.072	−0.060	−0.035	−0.050	−0.095 *
X9		0.044	0.040	0.031	0.143 **	0.039	0.073	0.035	0.078	0.056	0.043	0.077	0.111 **
X10		0.094 *	0.019	0.048	0.097 *	0.034	0.072	0.090 *	0.091 *	0.115 **	0.011	0.004	0.092 *
X11		0.072	0.115 **	0.069	0.102 *	0.086 *	0.126 **	0.077	0.068	0.051	0.047	0.070	0.070
X12		0.014	−0.030	0.002	0.056	−0.011	−0.004	0.017	0.000	−0.035	0.070	−0.031	−0.065
X13		0.022	0.091 *	0.130 **	0.179 **	0.113 **	0.118 **	0.025	0.148 **	0.070	0.109 **	0.094 *	0.030
X14		0.000	−0.009	0.011	0.010	−0.019	0.070	0.047	0.049	0.012	−0.086 *	0.038	0.023

***, **, and * represent significance levels of 1, 5, and 10%, respectively, variables M1–M12 are defined in Table 1, and X1–X14 are defined in Table 2.

**Table 5:** Preferences of residents for three types of urban greenways.

Greenway Type	Direct Preferences (*N* = 588)		Average Preference Score	
	Preference Number	Preference Frequency	Overall Average	Standard Deviation
Natural greenways	201	34.18%	4.066	0.872
Built greenways	143	24.32%	3.311	1.033
Mixed greenways	244	41.50%	4.112	0.949

**Table 6:** Greenway perception matrix of different resident groups.

	Survey Area	Natural Greenways	Built Greenways	Mixed Greenways	Overall Evaluation
Source of Respondents					
Natural greenways		4.066	3.791	3.485	3.781
Built greenways		3.454	3.311	3.352	3.372
Mixed greenways		3.694	3.679	4.112	3.828
Overall evaluation		3.738	3.594	3.650	-

**Table 7:** Importance–performance analysis quadrant placement of three types of urban greenways.

Category	Symbol	Variable	Natural Greenways	Built Greenways	Mixed Greenways
M1	Style	Traditional	Q1: Advantage area	Q3: Opportunity area	Q1: Advantage area
M2		Modern	Q3: Opportunity area	Q4: Improvement area	Q4: Improvement area
M3	Space	Sense of space	Q3: Opportunity area	Q4: Improvement area	Q4: Improvement area
M4		Sense of order	Q3: Opportunity area	Q4: Improvement area	Q4: Improvement area
M5	Color	Hue	Q4: Improvement area	Q1: Advantage area	Q4: Improvement area
M6		Color temperature	Q1: Advantage area	Q3: Opportunity area	Q4: Improvement area
M7	Environment	Greening coverage	Q1: Advantage area	Q2: Maintain area	Q1: Advantage area
M8		Greening facilities	Q1: Advantage area	Q1: Advantage area	Q1: Advantage area
M9	Psychology	Sense of belonging	Q1: Advantage area	Q1: Advantage area	Q1: Advantage area
M10		Sense of security	Q1: Advantage area	Q4: Improvement area	Q1: Advantage area
M11	Distance	From motorway	Q1: Advantage area	Q4: Improvement area	Q4: Improvement area
M12		From the Huangpu River	Q1: Advantage area	Q3: Opportunity area	Q1: Advantage area
Overall	-	-	Q1: Advantage area	Q4: Improvement area	Q1: Advantage area

## Appendix A. Questionnaire on the Public Perception of Huangpu Riverside Greenway. Place of Investigation (Filled in by Investigator)

**Table A1:** Please provide your personal basic information. (Please fill in the number of the corresponding option in the box).

**Item**	**1. Gender**	**2. Age**	**3. Education Status**	**4. Marital Status**	**5. Family Size**
Options	1 = Men 2 = Women	1 = Age 17 and under 2 = 18–29 years old 3 = 30–39 years old 4 = 40–49 years old 5 = 50–59 years old 6 = Age 60 and over	1 = Primary school and below 2 = Junior middle school 3 = High school 4 = Undergraduate 5 = Master’s degree and above	1 = Unmarried 2 = Married	1 = 1 person 2 = 2 persons 3 = 3 persons 4 = 4 persons 5 = 5 persons or more
Your choice					
**Item**	**6. Number of Elderly People**	**7. Number of Children**	**8. Shanghai Residence Registration Status**	**9. Residence in Shanghai**	**10. Average Working Hours Per Week**
Options	1 = 1 person or less 2 = 2 persons 3 = 3 persons or more	1 = 1 person or less 2 = 2 persons 3 = 3 persons or more	1 = Yes 2 = No	1 = <1 year 2 = 1–5 years 3 = 5–10 years 4 = 10–15 years 5 = 15 years or more	1 = <1 h 2 = 1–20 h 3 = 20–40 h 4 = 40–50 h 5 = >50 h
Your choice					
**Item**	**11. Per Capita Monthly Income**	**12. Main Modes of Transportation**	**13. Residential Area**	**14. Distance from Home to the Nearest Greenway**	
Options	1 = ¥4000 and below 2 = ¥4001–¥8000 3 = ¥8001–¥12,000 4 = ¥12,001–¥16,000 5 = ¥16,001–¥20,000 6 = >¥20,000	1 = Self-driving 2 = Walking 3 = Motorcycle and bicycle 4 = Taxi 5 = Bus and subway	1 = Within Inner Ring Road 2 = Inner–Middle Ring Road 3 = Middle–Outer Ring Road 4 = Outer Ring Road–The Ring Expressway 5 = Beyond the Ring Expressway	1 = <1 km 2 = 1–5 km 3 = 5–10 km 4 = >10 km	
Your choice					

**Table A2:** How do you rate the elements of Huangpu riverside greenway?

Symbol	Variable	Your Score (Please Tick the Corresponding Numbers)							Definition
Style	Traditional	Light	−2	−1	0	+1	+2	Heavy	Traditional degree of greenway
	Modern	Light	−2	−1	0	+1	+2	Heavy	Modern degree of greenway
Space	Sense of space	Enclosed	−2	−1	0	+1	+2	Wide	Spatial looseness and intensity of greenway
	Sense of order	Disordered	−2	−1	0	+1	+2	Ordered	Orderliness of greenway
Color	Hue	Dark	−2	−1	0	+1	+2	Clear	Brightness and contrast of greenway
	Color temperature	Cold	−2	−1	0	+1	+2	Warm	Overall cool and warm degree of greenway
Environment	Vegetation coverage	Low	−2	−1	0	+1	+2	High	Vegetation coverage of greenway
	Greening facilities	Imperfect	−2	−1	0	+1	+2	Perfect	Greening facilities along the greenway
Psychology	Sense of belonging	Unfamiliar	−2	−1	0	+1	+2	Familiar	Sense of belonging and familiarity of greenway
	Sense of security	Dangerous	−2	−1	0	+1	+2	Safe	Public security perception of greenway
Distance	From motorway	Far	−2	−1	0	+1	+2	Close	Distance between the greenway and the motorway
	From the Huangpu River	Far	−2	−1	0	+1	+2	Close	Distance between the greenway and the Huangpu River

The evaluation scale was classified into five levels with five intervals between each adjective combination. These intervals were symmetrical, with 0 as the midpoint. The interval values were −2, −1, 0, 1, and 2 from left to right, and these were used as the scoring method for the resident’s evaluations. The higher the score of each evaluation item, the more inclined the evaluation factor is to the right-hand adjective; the lower the score, the more inclined the evaluation factor is to the left-hand adjective.

**Table A3:** What is your preference for Huangpu riverside greenway? And how do you evaluate the importance and performance of Huangpu riverside greenway?

Greenway Type	Your favorite Type of Greenway	Comprehensive Evaluation	Importance Rating	Performance Rating
	(One choice from three, please check the box corresponding to the option)	(Please fill in the number of the corresponding option in the box) 1 = Poor 2 = Fair 3 = Good 4 = Very Good 5 = Excellent	(Please fill in the number of the corresponding option in the box) 1 = Not at All Important 2 = Somewhat Unimportant 3 = Neutral 4 = Somewhat Important 5 = Extremely Important	(Please fill in the number of the corresponding option in the box) 1 = Poor 2 = Fair 3 = Good 4 = Very Good 5 = Excellent
Natural greenways				
Built greenways				
Mixed greenways				
